# Depressive Symptoms and Posttraumatic Stress Disorder as Determinants of Preference Weights for Attributes of Obstetric Care among Ethiopian Women

**DOI:** 10.1371/journal.pone.0046788

**Published:** 2012-10-10

**Authors:** Magdalena M. Paczkowski, Margaret E. Kruk, Fasil Tessema, Ayalew Tegegn, Sandro Galea

**Affiliations:** 1 Department of Epidemiology, Mailman School of Public Health, Columbia University, New York City, New York, United States of America; 2 Department of Health Policy and Management, Mailman School of Public Health, Columbia University, New York, New York, United States of America; 3 Department of Epidemiology, College of Public Health and Medical Sciences, Jimma University, Jimma, Ethiopia; 4 Psychiatry and Behavioral Science Department, Howard University College of Medicine, Washington, District of Columbia, United States of America; Federal University of Rio de Janeiro, Brazil

## Abstract

**Background:**

Mental health, specifically mood/anxiety disorders, may be associated with value for health care attributes, but the association remains unclear. Examining the relation between mental health and attributes in a context where quality of care is low and exposure to suboptimal health conditions is increased, such as in Sub Saharan Africa (SSA), may elucidate the association.

**Methodology/Principal Findings:**

We assessed whether preference weights for obstetric care attributes varied by mental health among 1006 women from Jimma Zone, Ethiopia, using estimates obtained through a discrete choice experiment (DCE), a method used to elicit preferences. Facilities were described by several attributes including provider attitude and performance and drug/equipment availability. Mental health measures included depressive symptoms and posttraumatic stress disorder (PTSD). We used Bayesian models to estimate preference weights for attributes and linear models to investigate whether these weights were associated with mental health. We found that women with high depressive symptoms valued a positive provider attitude [β = −0.43 (95% CI: −0.66, −0.21)] and drug/equipment availability [β = −0.43 (95% CI: −0.78, −0.07)] less compared to women without high depressive symptoms. Similar results were obtained for PTSD. Upon adjusting for both conditions, value for drug/equipment availability was lower only among women with both conditions [β = −0.89 (95% CI −1.4, −0.42)].

**Conclusions/Significance:**

We found that women with psychopathology had lower preference weights for positive provider attitude and drug/equipment availability. Further work investigating why value for obstetric care attributes might vary by psychopathology in SSA is needed.

## Introduction

There are several well-documented factors that influence appropriate utilization of health facilities by the public. These include facility-related characteristics such as distance to facility, appropriate geographic distribution, waiting times, and costs as well as those that are provider-related such as provider type, level of training, and attitude [1]–[3]. Patient related characteristics such as sociodemographics, health status, past health seeking behavior, and preferences for the content and organization of health care also determine utilization of health services [1], [4]. Interest in eliciting patient preferences for health care has grown recently, primarily to clarify the type of health care experience patients desire in order to improve the responsiveness of health systems [5]–[12].

Several methods are available for eliciting health care preferences, including ranking and standard gamble. In recent years, conjoint analysis, which refers to a group of several approaches used to elicit preferences, has become a popular method [13], [14]. One approach, the discrete choice experiment (DCE), is based on describing health care services or products with a set of characteristics, or attributes, that are themselves defined by levels [5], [6], [13]. Respondents are shown scenarios that bring together several attributes that describe a particular service, for example, a health care clinic, and choose their preferred scenario. The choices of individuals then permit estimation of the value and importance of each attribute. DCEs have shown good consistency with other methods, and good validity and test-retest reliability [15]. They are useful to understand what a patient population expects from a service and can guide health care professionals in planning future health care needs.

Studies focused on estimating patients’ value of health care attributes and their levels have been conducted for general health care, cancer and cardiovascular care, and labor and delivery, among others [6]–[8], [16]–[19]. A few studies have assessed whether value of health care attributes is associated with individual characteristics. These extant studies have shown that value can be influenced by factors such as demographics, physical, and mental health [3], [8], [20]. The latter factor, mental health, is salient for two central reasons. First, those with poor mental health may be more likely to value certain attributes less compared to those with better mental health. In particular, those with mood/anxiety disorders may derive less satisfaction from consumption, especially when the disorders are severe [21]. This association may stem from symptoms of depressed mood and anhedonia, highly prevalent in these disorders [22]–[25]. Second, the global burden of disability from psychopathology, specifically unipolar depressive disorders, was the fourth-leading cause of disability worldwide in 2002, and by 2030 is projected to become the second cause of disability worldwide [26]. This may translate to a greater need for health care tailored to those with psychopathology.

A few studies have explored the association between mental health and value of health care attributes, mainly in high-income countries. Some studies have shown that individuals with mental illness do exhibit different preferences for general health care compared to those without mental illness [20], [27]. Arora et al. reported that persons with depression were more likely to prefer shared decision making with a health care provider compared to those without depression [20]. Walters et al. found that those with mild or moderate mental distress were more likely to prefer help from informal sources as well as from a general practitioner rather than mental health oriented therapies [27]. Alternatively, in a study of Liberian men and women, Kruk et al. found null associations between PTSD and health care preferences [18]. Thus, the association between mental health and value of health care attributes remains unclear.

There is a paucity of studies, however, that have assessed the relation between mental health and value for health care attributes in low-income countries. The lack of studies assessing the relation between mental health and value in low-income countries, especially in Sub Saharan Africa (SSA), is problematic. Health care structure, quality, and access in SSA contexts differs substantially compared to high-income countries. In certain countries, such as Ethiopia, care may be accessible in most areas, but low investment in health care leads to low quality of care. Additionally, populations in SSA may experience increased exposure to factors such as poor economic conditions, poor physical health, stressful life events, and violent or shocking traumatic events, which are associated with mood/anxiety disorders [28], [29]. A context where low quality care is an explicit, persistent problem and where health and social conditions are suboptimal provides a unique opportunity to shed light on the role that mental health problems, specifically mood/anxiety disorders, play in value of health care attributes.

Kruk et al. conducted a discrete choice experiment (DCE) to elicit preferences for delivery care in a cohort of Ethiopian women [19]. They found that having a doctor as a provider and a facility well-stocked with medicine and equipment, both patient-discernable proxies for quality of care, were the two most valued aspects in selecting a clinic for delivery [19]. To investigate the association between mental health and value of attributes for health care, we assessed whether value of obstetric care attributes differed based on depressive symptoms and posttraumatic stress disorder (PTSD), two of the commonest mood/anxiety disorders, in a cohort of Ethiopian women from Jimma Zone, Oromia Regional state [30].

## Methods

### Ethics Statement

Trained interviewers fully explained the purpose, process, benefits, and risks to all potential study participants before consent was obtained. Participation was voluntary. Written informed consent was obtained from all participants. The Institutional Review Boards of the University of Michigan and Jimma University reviewed and approved the study protocol.

### Study Area and Sample

This study took place in Jimma zone, Southwest Ethiopia in the Gilgel Gibe Field Research Center outside of Jimma Town. The Gilgel Gibe Growth and Development Study is a longitudinal cohort study of families in the Gilgel Gibe Field Research Center that has been previously described elsewhere [31], [32]. Briefly, the study collected questionnaire and anthropometric information, which refer to measurements of weight and height, from parents, and conducted developmental assessments on their children in December – January 2006/2007 [33]. The second wave of the study, presented here, added questions on health care utilization as well as a discrete choice experiment on health care utilization. This analysis used data from 1006 women who were included in the second wave of the study with a response of 85%. Data was collected between May and August 2007.

### Instrument Design and Fielding

A structured questionnaire and consent document were developed in English and translated and back translated into the two dominant languages in the study area: Amharic and AffanOromifa by native speakers. Men and women were interviewed separately by trained interviewers. All respondents were interviewed directly by the interviewer for the duration of the survey and all survey information obtained was recorded by the interviewer. The structured questionnaire obtained information about (a) demographic and anthropometric characteristics of the women, (b) household wealth, (c) parity and health care utilization, (d) depressive symptoms, and (e) PTSD.

### Demographic and Anthropometric Characteristics

Information was collected on age, formal years of education, and weight and height. Anthropometric measures, meaning weight and height, were used to calculate BMI. BMI was dichotomized to obtain chronic energy deficiency (CED), used as a measure of physical health [33]–[35]. Women with a BMI less than or equal to 18.5 were considered to have CED [36].

### Household Wealth

Household wealth was measured using a summary indicator composed of material assets and livestock ownership, both good measures of permanent wealth in low-income countries [37]. Both asset types were summed and the scores were dichotomized based on the median. The final summary indicator was created by grouping households with low material goods and livestock ownership as “low" wealth, households with at least high material goods or livestock ownership as “medium" wealth, and households with both high material goods and livestock ownership as “high" wealth.

### Parity and Facility Utilization for Delivery

Women were asked about parity, referring to the total number of times a woman has given birth, where they delivered their most recent child (home, health post, health center, or hospital), and how many children overall were delivered in a health facility.

### Depressive Symptoms

We used the Hopkins Symptom Checklist, a validated inventory of 15 symptoms of depression to measure depressive symptoms [38]–[40]. Respondents were asked how much they were bothered by the set of symptoms in the past week. Responses were on a four point likert scale, and ranged from “not at all" to “extremely". From the Hopkins Symptom Checklist we calculated a measure of depressive symptoms by summing across each item to obtain a total score. We created a dichotomous form of depressive symptoms, following established protocols and cut-offs. We divided the total depressive symptom score by the number of items, 15, and considered scores above 1.75 as evidence of high symptoms of depression [38]–[40].

### Posttraumatic Stress Disorder

We assessed PTSD symptoms using a 16-item Harvard Trauma Questionnaire inventory based on Diagnostic and Statistical Manual of Mental Disorders IV PTSD criteria [39], [41]. Respondents were asked how much they were bothered by the set of symptoms in the past week. Responses were on a four point likert scale, and ranged from “not at all" to “extremely". We obtained the total score by summing across each item and followed established protocols and cut-offs to create a dichotomous indicator of PTSD by dividing the total score by the number of items, 16, and considered scores above 2.0 as “checklist positive" for PTSD [41].

### Discrete Choice Experiment

We designed the DCE to estimate the relative value of health facility features to women in considering delivery of their next child. It has been described elsewhere [19], [42]. Briefly, based on qualitative and quantitative literature, including the 2005 Ethiopia Demographic and Health Survey, and discussions with local health providers, we selected key attributes of the service that included facility distance (in hours), cost (in Ethiopian Birr), provider type (doctor, nurse, health extension worker), availability of transportation to the facility, drug/equipment availability, and provider attitude (whether the provider smiles and listens) [43]–[45]. We assigned levels to each attribute, identified scenarios to present, and fielded the experiment using trained interviewers to obtain preferences. During the DCE, we presented respondents with three options: facility A or B, described by the attributes listed above, and a third option C, representing the neither or opt-out option. We informed respondents that each option described two possible health facilities and to imagine that they were deciding where to deliver their next baby. Respondents were asked to indicate which of the two facilities they preferred to use for their future delivery or, if they did not want to use either facility, to indicate neither or none. A sample card used for this experiment has been previously published [19].

### Statistical Analysis

Descriptive statistics were calculated for the study sample using SAS software [46]. We used a Student’s t test for the difference of means to assess whether preference weights differed by high depressive symptoms and PTSD status. P-values were used to assess significance for all t tests. Generalized estimating equations (GEE), which yield robust standard errors, were used to assess the relation between mental health (PTSD and depressive symptoms) and the continuous outcomes, individual-level preference weights for health facility attribute levels, in models that were adjusted for age, formal years of education, household wealth, and chronic energy deficiency. Significance of associations between explanatory variables and outcomes were assessed using 95% confidence intervals. All GEEs were modeled using SAS [46].

The process for obtaining our individual level preference weights for health facility attribute levels consisted of two steps. First, we estimated the individual weights, or coefficients, for each attribute level using Hierarchical Bayesian procedures via Sawtooth Software’s Choice Based Conjoint/Hierarchical Bayes statistical program [47]. Transport availability, drug/equipment availability, and provider attitude were treated as dichotomous variables, provider type was treated as a categorical variable, while cost and distance were treated as continuous variables. All dichotomous and categorical variables were dummy coded variables. The estimation technique has been described elsewhere [19], [42]. Briefly, the Bayesian procedure estimates the distribution of coefficients across the population and combines that with the individual’s coefficients to derive posterior or conditional estimates of the individual’s coefficients. Conceptually, the use of Hierarchical Bayes modeling permits individual level estimates by borrowing information from the group and adjusts for lack of respondent consistency across choice cards by weighting down the influence of inconsistent respondents [48]. The model output consists of individual-level coefficients for attribute levels for each respondent and the average coefficients for attribute levels for the sample. Comparing attribute levels, larger preference weights denote a higher value for one attribute level compared to another.

Second, we exported the individual preference weights for the attribute levels–a measure of value for that attribute level – into SAS to serve as continuous outcomes for the GEE models. Each preference weight represents an interval value expressing the gain or loss in utility for moving from one level to another. Therefore, the preference weight for doctor is the value gained in moving from a nurse to a doctor, whereas the preference weight for nurse is the value of moving from a health extension worker to a nurse. The beta coefficients from the GEEs are a function of both individual preference weights for each attribute as it relates to determining facility choice, as well as the structure of the discrete choice experiment, specifically, the number of levels included to define each attribute. Therefore, the magnitudes are not readily interpretable because they reflect an arbitrary scale. However, the direction and significance of the beta coefficients indicate whether a women is more or less likely to value a particular attribute or level of an attribute based on an explanatory variable. The ratios of two beta coefficients are also interpretable and indicate the marginal rate of substitution (MRS), or relative importance of one attribute compared to another.

The funders did not have any role in the design, collection, or analysis of this study.

## Results

Complete data were available for 1006 women. The characteristics of the respondents are shown in Table 1. The mean age was 26.5 (SD 5.5), while the mean years of formal education were 0.67 (SD 2.0) and 42% were of low wealth. The mean number of total children was 3.4 (SD 1.8) but the mean number of children born in a health facility was lower, at 0.1 (SD 0.4). Similarly, 93.8% of women who had a pregnancy in the last five years did not give birth in a facility. The prevalence of CED was 23.9% while the prevalence of high depressive symptoms and PTSD was 22.4% and 14.1%, respectively. The Pearson correlation between depressive and PTSD symptoms was 0.71 (p<0.0001). Among those who had PTSD, 78.2% also had high depressive symptoms, while, among those with high depressive symptoms, 49.3% had PTSD. Overall, 11.04% of women endorsed both conditions.

**Table 1:** Characteristics of a sample of women from Jimma Zone, Oromia, Ethiopia.*

Characteristics	n = 1006†
Age, mean (SD)	26.5 (5.5)
Years of education, mean (SD)	0.67 (2.0)
Number of total living children, mean (SD)	3.4 (1.8)
Number of children born in a health facility, mean (SD)	0.1 (0.4)
Place of last delivery‡	
Home§	942 (93.8)
Health post	10 (1.0)
Health center	44 (4.4)
Hospital	7 (0.7)
Socioeconomic status¶	
Low	395 (42.0)
Medium	497 (52.9)
High	48 (5.1)
Has chronic energy deficiency**	240 (23.9)
Has high depressive symptoms††	225 (22.4)
Has PTSD‡‡	142 (14.1)

* Data is n (%) unless otherwise specified.

† Totals may not add up to 1006 due to missing data.

‡ One woman delivered in an unspecified location.

§ Home includes respondent’s or someone else’s home.

¶ Households were divided into low, medium, or high wealth based on material goods and live stock holdings.

** Body Mass Index <18.5.

†† Hopkins Symptom Checklist >1.75.

‡‡ Harvard Trauma Questionnaire >2.0.

The means and standard deviations of the preference weights, stratified by high depressive symptoms and PTSD, are shown in Table 2. Means of preference weights for attributes and levels were all positive, with the exception of cost, indicating that, on average, regardless of mental health status, these attributes and levels were positively associated with preference for facility. However, women with high depressive symptoms or PTSD had lower average preference weights compared to women without these conditions, with the exception of transportation availability, where the average preference weight was higher for women with either condition compared to women without. Mean differences in preference weights between women with high depressive symptoms and women without were significant for nurses as provider type, provider attitude, and drug/equipment availability. Similarly, mean differences in preference weights between women with PTSD and women without were significant for doctors or nurses as providers, provider attitude, drug/equipment availability and transport.

**Table 2:** Average preference weights, stratified by depressive symptoms and PTSD.*

	High depressive symptoms†		PTSD‡	
Preference weight	Yes (n = 225)	No (n = 781)	Yes (n = 142)	No (n = 863)
Provider type				
Doctor	2.0 (1.5)	2.1 (2.0)	1.8 (1.4) §	2.2 (1.9)
Nurse	1.5 (1.3) §	1.7 (1.7)	1.4 (1.2) §	1.7 (1.6)
Provider attitude	1.1 (1.2) §	1.6 (1.6)	0.93 (1.1) §	1.5 (1.6)
Drug/equipment availability	3.5 (2.3) §	4.0 (2.4)	3.1 (2.2) §	4.0 (2.3)
Transport availability	0.53 (0.89)	0.43 (0.84)	0.61 (1.0) §	0.42 (0.83)
Cost	−.29 (0.56)	−0.29 (0.57)	−0.27 (0.60)	−0.29 (0.56)
Distance	0.15 (0.45)	0.18 (0.42)	0.16 (0.45)	0.17 (0.42)

* Data is mean (SD).

† As measured by the Hopkins Symptom Checklist.

‡ As measured by the Harvard Trauma Questionnaire.

§ Mean difference significant at p<0.05.

Table 3 shows multivariable models with high depressive symptoms as the main explanatory variable. Women with high depressive symptoms had a lower preference weight for having a provider with a positive attitude compared to women without high depressive symptoms [β = −0.43, 95% CI: −0.66, −0.21]. Women with high depressive symptoms also had a lower preference weight for drug availability in facility compared to women without high symptoms [β = −0.43, 95% CI: −0.78, −0.07]. Depressive symptoms were not significantly associated with having a doctor or nurse as a provider, transport availability to a facility, cost, or distance. All models were adjusted for age, formal years of education, wealth, and CED. Lastly, for women with high depressive symptoms, the MRS between provider attitude and drug/equipment availability was 1.0, indicating that neither attribute has more weight for the value of a health facility than the other.

**Table 3:** Multivariable associations between individual level attribute preference weights, high depressive symptoms and post traumatic stress disorder.

	Doctor	Nurse	Positive provider attitude	Drugs available	Transport available	Cost	Distance
	β (95% CI)	β (95% CI)	β (95% CI)	β (95% CI)	β (95% CI)	β (95% CI)	β (95% CI)
Multivariable association between preference weights and high depressive symptoms*							
High depressive symptoms†	−0.06 (−0.33, 0.20)	−0.15 (−0.38, 0.08)	−0.43 (−0.66, −0.21)	−0.43 (−0.78, −0.07)	0.09 (−0.04, 0.22)	0.03 (−0.05, 0.12)	−0.024 (−0.09, 0.04)
Multivariable association between preference weights and post traumatic stress disorder*							
Posttraumatic stress disorder‡	−0.28 (−0.60, 0.03)	−0.19 (−0.47, 0.08)	−0.51 (−0.78, −0.24)	−0.84 (−1.3, −0.42)	0.16 (0.007, 0.31)	0.05 (−0.05, 0.15)	−0.006 (−0.08, 0.07)
Multivariable association between preference weights, high depressive symptoms, and post traumatic stress disorder*							
No high depressive symptoms†,no PTSD‡	Ref	Ref	Ref	Ref	Ref	Ref	Ref
High depressive symptoms†,no PTSD‡	−0.02 (−0.36, 0.33)	−0.17 (−0.47, 0.13)	−0.42 (−0.71, −0.12)	−0.008 (−0.47, 0.46)	0.07 (−0.10, 0.24)	−0.02 (−0.13, 0.09)	0.007 (−0.08, 0.09)
No high depressive symptoms†,PTSD‡	−0.69 (−1.3, −0.06)	−0.39 (−0.94, 0.16)	−0.70 (−1.2, −0.17)	−0.41 (−1.3, 0.44)	0.25 (−0.06, 0.56)	−0.08 (−0.29, 0.12)	0.13 (−0.02, 0.29)
High depressive symptoms†,PTSD‡	−0.17 (−0.52, 0.18)	−0.18 (−0.49, 0.13)	−0.52 (−0.82, −0.22)	−0.89 (−1.4, −0.42)	0.16 (−0.02, 0.33)	0.08 (−0.03, 0.20)	−0.04 (−0.13, 0.04)

* Model adjusted for age, education, SES, and chronic energy deficiency.

† Hopkins Symptom Checklist >1.75.

‡ Harvard Trauma Questionnaire >2.0.

The multivariable models including PTSD as the main covariate of interest are shown in Table 3. Women with PTSD had a lower preference weight for having a provider with a positive attitude [β = −0.51, 95% CI: −0.78, −0.24] compared with women who did not have PTSD. Women with PTSD also had a lower weight for drug availability [β = −0.84, 95% CI: −1.3, −0.42] compared to women who did not have PTSD. PTSD was positively associated with transport availability to a facility [β = 0.16, 95% CI: 0.007, 0.31]; women with PTSD had a higher weight for transport availability compared to women without PTSD. PTSD was not significantly associated with preference for having a doctor or a nurse as a provider, cost, or distance. All models were adjusted for age, formal years of education, wealth, and CED. Lastly, for women with PTSD, the MRS between provider attitude and drug/equipment availability was 0.61, indicating that positive provider attitude had a lower negative effect on the value of a health facility compared to drug equipment availability.

The multivariable models including both PTSD and depressive symptoms simultaneously are shown in Table 3. Women with high depressive symptoms but no PTSD [β = −0.42, 95% CI: −0.71, −0.12], PTSD but no high depressive symptoms [β = −0.70, 95% CI: −1.2, −0.17], and both conditions [β = −0.52, 95% CI: −0.82, −0.22] had lower preference weights for provider attitude compared to women with neither conditions. Having only high symptoms of depression or only PTSD was not significantly associated with the weights for drug availability while having both PTSD and high depressive symptoms was significantly associated with drug availability [β = −0.89, 95% CI: −1.4, −0.42]. Transport availability was no longer significantly associated with PTSD upon adjusting for high symptoms of depression while having a doctor as a provider was significantly associated with having only PTSD [β = −0.69, 95% CI: −1.3, −0.06]. All models were adjusted for age, formal years of education, wealth, and CED. Lastly, the MRS between positive provider attitude and drug/equipment availability was 52.5, 1.71, and 0.584 among women with only high depressive symptoms, women with only PTSD, and women with both conditions, respectively.

## Discussion

We found that preference weights for two out of six attributes of obstetric care among a cohort of Ethiopian women from the Gilgel Gibe Field Research Center were influenced by measures of mental health, adjusted for physical health and demographics. We found that women with high depressive symptoms or PTSD did not value a provider with good interpersonal skills and clinics with consistent drug/equipment availability as much as women without high depressive symptoms or PTSD in adjusted models. In models adjusted for both conditions, the association between poor mental health and drug/equipment availability persisted only among women with both high depressive symptoms and PTSD.

Our results pertaining to differences in preference weights based on depressive symptoms and PTSD are consistent with a growing body of literature that recognizes that value of health care aspects can vary based on psychopathology. Depression may be associated with lack of trust, overall and for health care providers [49]. Lee et al. showed that in a population of adult patients with type 2 diabetes, those with poorer mental health reported lower trust in physicians compared to those with better mental health in models adjusted for age, sex, and duration of diabetes [49]. Therefore, it is possible that women with high depressive symptoms or PTSD had lower trust in providers, which in turn led to less concern about their interpersonal skills. It is also likely that pessimism and anhedonia, common symptoms of poor mental health, led women to care less about provider interpersonal skills compared to women without mental illness [22], [23].

That women with high depressive symptoms and PTSD prefer drug/equipment availability less than women without these illnesses may also be partially explained by symptoms present in several mental disorders including impaired cognition, pessimism, and anhedonia [22]–[25], [50]. It is possible that women without high depressive symptoms and PTSD were more capable of making the cognitive link between better availability of drugs and equipment and the ability to intervene in the case of an obstetric emergency [50]. It may also be the case that women with PTSD and high depressive symptoms are more apathetic or resigned to the potential for poor obstetric outcomes and therefore do not value potentially effective clinic inputs [51]. This is reinforced by our finding of lower preference weights among women with PTSD for more skilled providers: doctors and nurses. In contrast, women with PTSD valued the availability of transport more than women without PTSD, though the association was no longer significant in models adjusted for depressive symptoms, suggesting that reducing the logistic effort is important to women who may already feel overwhelmed.

While high depressive symptoms and PTSD were associated with the preference weight for drug/equipment availability, in models adjusted for mutually exclusive categories of both conditions, only women with both high depressive symptoms and PTSD had a lower weight compared to women with neither condition. This suggests that symptoms of pessimism, anhedonia, and others related to depression may not have a significant influence over cognitive links and resignation as discussed above without the presence of symptoms of PTSD. It may be that women who endorse both conditions suffer more impairment compared to women that endorse only one and this diminishes their ability to foresee individual benefits of potentially life saving interventions.

It is not surprising that we found similar associations between preference weights and high depressive symptoms and PTSD considering the symptom overlap between these disorders [52], [53]. Specifically, problems concentrating and sleeping, and anhedonia characterize both disorders [53]. In our population, 45.6% and 40.3% of women with high depressive symptoms were bothered very much or extremely with sleep problems and anhedonia, respectively, while 44.4% and 52.1% of women PTSD reported being bothered quite a bit or extremely with sleep and anhedonia. This suggests that symptom overlap, particularly anhedonia, may partially explain similar associations between preference weights and both disorders.

Our results need to be interpreted in light of several limitations. First, neither the Hopkins Symptom Checklist nor the Harvard Trauma Questionnaire has been formally validated in our specific population. However, both have been used in a variety of cultures and settings, including in low-income countries, which alleviates concerns regarding validity [38]–[40]. Second, it is difficult to draw inferences regarding the magnitudes of the coefficients from the GEEs due to dependence on the structure of the discrete choice experiment and therefore, we are unable to quantify and compare demand between women with psychopathology and women without psychopathology. Third, while we report positive findings between transport availability and PTSD, and null findings between PTSD, depressive symptoms, and distance to a facility, it is likely that both attributes are dependent on one another, and thus, due to confounding, it is not possible to isolate the associations between psychopathology, transportation availability, and distance. Fourth, the findings presented here are limited by the design of the discrete choice experiment. Although we used the literature and health professionals to identify the attributes and levels that were the most appropriate, it is possible that we overlooked others important for facility description. Additionally, although we allowed respondents to choose “neither" as a possible scenario, this was not a true opt-out, as most women in this population deliver at home rather than at a facility. Thus, it is possible that women, specifically those with poor mental health, felt less motivated to choose between scenarios that they did not see as realistic delivery options. This may partially explain the lower preference weights for quality related attributes among women of poor mental health. Fifth, this study was conducted in a specific population of rural Ethiopia women and therefore the findings reported here might not be generalizable to other populations.

These limitations notwithstanding, we found that preference weights for delivery care among women varied based on mental health as well as other demographic and health variables. Our results indicate that women who have high depressive symptoms or posttraumatic stress disorder are less influenced by quality of care characteristics in selecting facilities for childbirth. This implies that these women are less likely to increase their use of health facilities for maternal health care even as the health system improves. More work is needed to understand whether women with psychopathology utilize high quality facilities, or facilities overall, differently compared to women without. However, it is important for health practitioners to be aware that women with psychopathology may not be aware of the beneficial effect of higher quality facilities on their obstetric outcomes, likely due to the influence of anhedonia, depressed mood, and other symptoms characteristic of mood/anxiety disorders. In addition to the self-evident imperative to step up efforts to identify and treat depressive symptoms and PTSD in rural Ethiopia, this suggests that need for more concerted outreach to pregnant women with psychopathology to encourage facility utilization. More generally, our findings highlight the importance of women’s mental health in understanding variations in value for aspects of obstetric care in low-income countries.
